# Computational Simulation of HIV Protease Inhibitors to the Main Protease (Mpro) of SARS-CoV-2: Implications for COVID-19 Drugs Design

**DOI:** 10.3390/molecules26237385

**Published:** 2021-12-05

**Authors:** Wei Yu, Xiaomin Wu, Yizhen Zhao, Chun Chen, Zhiwei Yang, Xiaochun Zhang, Jiayi Ren, Yueming Wang, Changwen Wu, Chengming Li, Rongfeng Chen, Xiaoli Wang, Weihong Zheng, Huaxin Liao, Xiaohui Yuan

**Affiliations:** 1 Institute of Biomedicine, Jinan University, Guangzhou 510632, China; wei926yu@163.com (W.Y.); chenchun_jnu@163.com (C.C.); ws_wym@126.com (Y.W.); swcw965@163.com (C.W.); cli816@163.com (C.L.); 2 MOE Key Laboratory for Nonequilibrium Synthesis and Modulation of Condensed Matter, School of Physics, Xi’an Jiaotong University, Xi’an 710049, China; zyz9856@stu.xjtu.edu.cn (Y.Z.); yzws-123@xjtu.edu.cn (Z.Y.); 3 Anhui Province Key Laboratory of Pollutant Sensitive Materials and Environmental Remediation, College of Life Sciences, Huaibei Normal University, Huaibei 235000, China; dolphin1009@sina.com (X.W.); zxcyfr@163.com (X.Z.); 4 Zhuhai College of Science and Technology, Zhuhai 519041, China; jiayi80@126.com; 5 Zhuhai Trinomab Biotechnology Co., Ltd., Zhuhai 519040, China; chenrongfeng2013@163.com (R.C.); bolinjiari@163.com (X.W.); zwh13901154411@139.com (W.Z.)

**Keywords:** COVID-19, SARS-CoV-2, main protease (Mpro), HIV protease inhibitor, docking, molecular dynamics (MD) simulation

## Abstract

SARS-CoV-2 is highly homologous to SARS-CoV. To date, the main protease (Mpro) of SARS-CoV-2 is regarded as an important drug target for the treatment of Coronavirus Disease 2019 (COVID-19). Some experiments confirmed that several HIV protease inhibitors present the inhibitory effects on the replication of SARS-CoV-2 by inhibiting Mpro. However, the mechanism of action has still not been studied very clearly. In this work, the interaction mechanism of four HIV protease inhibitors Darunavir (DRV), Lopinavir (LPV), Nelfinavir (NFV), and Ritonavire (RTV) targeting SARS-CoV-2 Mpro was explored by applying docking, molecular dynamics (MD) simulations, and MM–GBSA methods using the broad-spectrum antiviral drug Ribavirin (RBV) as the negative and nonspecific control. Our results revealed that LPV, RTV, and NFV have higher binding affinities with Mpro, and they all interact with catalytic residues His41 and the other two key amino acids Met49 and Met165. Pharmacophore model analysis further revealed that the aromatic ring, hydrogen bond donor, and hydrophobic group are the essential infrastructure of Mpro inhibitors. Overall, this study applied computational simulation methods to study the interaction mechanism of HIV-1 protease inhibitors with SARS-CoV-2 Mpro, and the findings provide useful insights for the development of novel anti-SARS-CoV-2 agents for the treatment of COVID-19.

## 1. Introduction

Currently, Coronavirus Disease 2019 (COVID-19) caused by a new type of coronavirus (SARS-CoV-2) infection is spreading globally and has posed a significant threat to the human health and economic stability over the world [1,2]. SARS-CoV-2 and SARS-CoV have a very high homology, with the ability of spreading from person to person through respiratory droplet transmission and contact transmission. After infection, they can attack vascular endothelial cells, epithelial cells, and immune cells, resulting in severe acute respiratory syndrome. It can take months or even years to develop new specific treatments. Based on data from the World Health Organization, at least 24 novel vaccines and at least 10 neutralizing antibody cocktail drugs have entered the clinical research phase, while several COVID19 vaccines have been approved for emergency use in several countries across continents. In November 2021, the UK took the lead in announcing the approval to use the COVID-19 drug Molnupiravir developed by Merck, as a ribonucleotide analog, which replaces normal ribonucleotides when the virus replicates, thereby preventing virus replication. In addition, there are many drugs in the clinical and preclinical stages around the world. The development of new drugs against SARS-CoV-2 or SARS-CoV at the same time will still be a significant challenge for mankind for a long period of time in the future.

**Figure 1:**
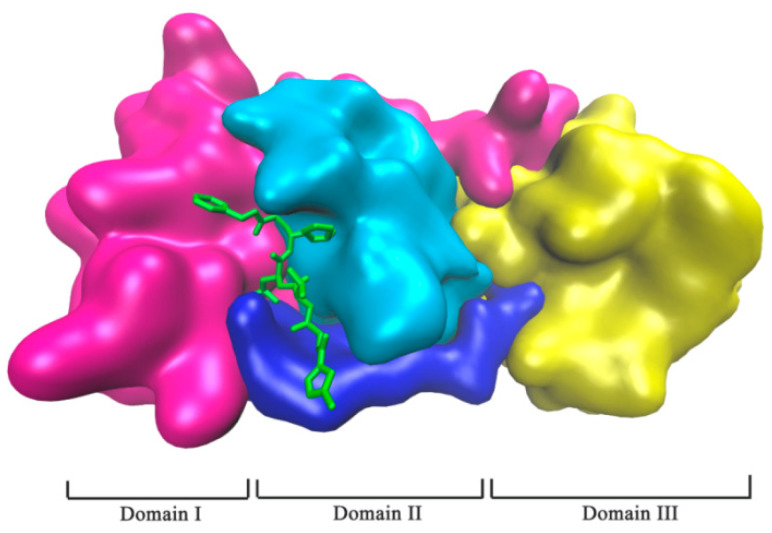
SARS CoV-2 Mpro in complex with protease inhibitor N3 (PDB: 6LU7). Mpro of SARS-CoV-2 was shown in domains: Domain I (in magenta), Domain II (in cyan), and Domain III (in yellow), and the linker region (in blue). Protease inhibitor N3 is represented in the green stick model.

The main protease (Mpro) of SARS-CoV-2 as a protease is one of the most attractive targets, which is involved in the virus replication process [3,4]. Mpro is a homodimer of two protomers, and each protomer is composed of three domains (I, II, and III), as shown in Figure 1 (PDB: 6LU7). It has atypical Cys-His doublets (His41 and Cys145) in the gap between domain I and II, which are two catalytical sites of Mpro [5,6]. The maturation process of the virus is highly dependent on Mpro, in which it can cleave the polyprotein body produced by the transcription of viral genomic RNA in host cells to produce key proteins required for virus replication, such as RNA-dependent RNA polymerase (RdRp, Nsp12) and helicase (Nsp13) [7]. Thus, the inhibition of Mpro can prevent the replication of the virus [8,9,10]. Therefore, Mpro is an ideal drug target for the treatment of COVID-19.

It was found that the protease activity of HIV is similar between SARS-CoV and SARS-CoV-2 [11], and HIV protease inhibitors could hinder the replication of SARS-CoV virus [12,13,14,15]. In attempts to screen anti-SARS-CoV drugs, it was revealed that many potential drugs identified by molecular simulation or other experimental methods were HIV protease inhibitors in spite of the structures of Mpro and HIV protease being different. In addition, the SARS-CoV-2 and SARS-CoV are closely related, with an amino acid sequence similarity of 96% [16], and their activity pockets are very similar. Therefore, it can be speculated that HIV protease inhibitors may be equally effective against both SARS-CoV-2 and SARS-CoV. Actually, in vitro activity data have indicated the virus suppression abilities of HIV protease inhibitors, although these inhibitors may not be used as inhibitors for SARS-CoV-2 Mpro, but laid an important foundation for the development of new anti-SARS-CoV-2 small-molecule inhibitor drugs for clinical applications [17].

**Figure 2:**
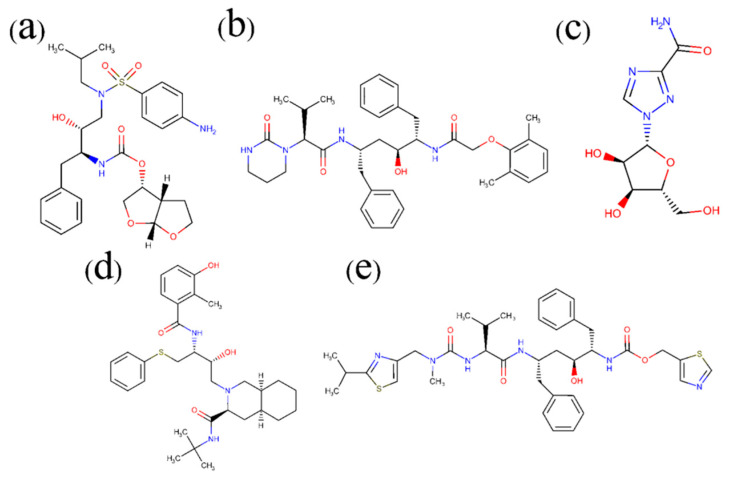
Chemical structures of common drugs used in this work: Darunavir (DRV, (**a**)) Lopinavir (LPV, (**b**)), Ribavirin (RBV, (**c**)), Nelfinavir (NFV, (**d**)), and Ritonavir (RTV, (**e**)).

Some reports have revealed that Nelfinavir (NFV), Lopinavir (LPV), Darunavir (DRV), and Ritonavir (RTV) as routine medicines for the treatment of HIV-1 infection [11,18,19,20] may be effective against COVID-19 [21,22,23]. In fact, RTV and LPV have been attempted in clinic use, and the therapeutic effect is under further clinical observation. Due to the ongoing pandemic of the COVID-19 outbreak, there is an urgent need to advance our understanding of the binding effects of common HIV protease inhibitors with SARS-CoV-2, but the mode of the molecular interaction between these drugs and SARS-CoV-2 is still unclear. According to the previous experimental data, in this work, docking calculation, molecular dynamics (MD) simulation, molecular mechanics–generalized Born surface area (MM–GBSA), and computational alanine scanning (CAS) methods were applied in combination to study the interaction mechanisms between four inhibitors and the main protease (Mpro) of SARS-CoV-2. Meanwhile, the broad-spectrum antiviral drug Ribavirin (RBV) was included in the simulation process as a control (Figure 2). Finally, the pharmacophore model was used to analyze the structural commonality and mechanism of action for Mpro small-molecule inhibitors.

Our study might be beneficial for the molecular design for the development of new small-molecule drugs as lead compounds as specific treatments for COVID-19.

## 2. Results and Discussion

### 2.1. Complex Formation by Docking

**Figure 3:**
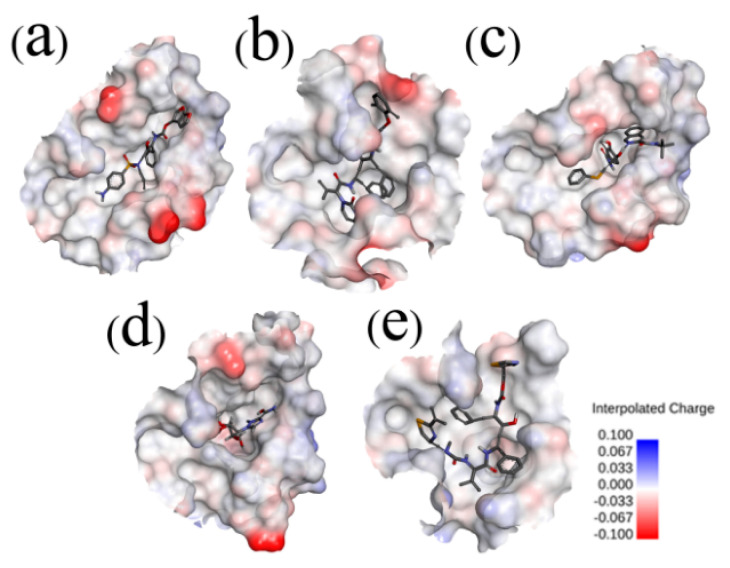
Electrostatic potential surfaces of different inhibitors with substrate-binding pocket of SARS-CoV-2 Mpro: SARS-CoV-2 Mpro-DRV (**a**), SARS-CoV-2 Mpro-LPV (**b**), SARS-CoV-2 Mpro-NFV (**c**), SARS-CoV-2 Mpro-RBV (**d**), and SARS-CoV-2 Mpro-RTV (**e**). Red shows negative charge and blue shows positive charge. Most of the substrate-binding pocket is net neutral and facilitates the inhibitor binding. However, in the SARS-CoV-2 Mpro-RBV complex, the substrate-binding pocket shows negative charge. The Connolly surface of the protein was created using the Discovery Studio scripts with surface electrostatic potential.

**Figure 4:**
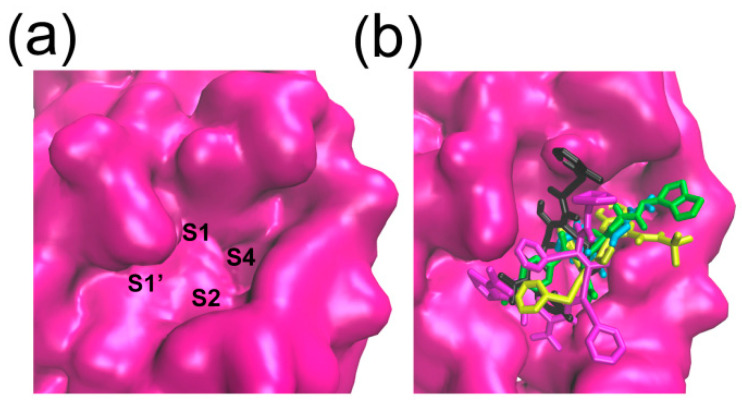
Selected hits clustered in SARS-CoV-2 Mpro. The surface representation of protein (in pink) is created using the Discovery Studio scripts. The S1, S2, S4, and S1’ substrate-binding pockets are labeled (**a**). The five Small molecular ligands superposed at conserved substrate-binding pockets of SARS-CoV-2 Mpro (**b**).

The crystal structure of SARS-CoV-2 Mpro in complex with ligand N3 (PDB: 6LU7) was retrieved from the RCSB PDB database and used in the docking process [24]. The binding site was assigned according to the location of ligand N3, and the redocked pose of N3 with SARS-CoV-2 Mpro was in a manner consistent with the crystallographic pose (PDB: 6LU7), with the RMSD (root-mean-square deviation) value being less than 0.200 nm. The successful redocking of N3 confirmed that the docking process is reliable and able to emulate the binding poses of Nelfinavir (NFV), Lopinavir (LPV), Darunavir (DRV), Ritonavir (RTV), and Ribavirin (RBV). Subsequently, the assemble-based docking was performed, and the docking results are summarized in Figure 3 and Figure 4. Note that interaction energy (*E_int_*) refers to the receptor–ligand interaction energy, and total energy (*E_total_*) includes *E_int_* and the internal ligand strain energy. It was found that LPV, RTV, and NFV are candidates, with *E_total_* values being smaller than −141.17 kJ/mol. The *E_total_* of DRV with SARS-CoV-2 Mpro is calculated to be −122.21 kJ/mol, while the binding of RBV is generally mismatched (*E_total_* = −44.14 kJ/mol), and the value of ligand N3 is −331.08 kJ/mol. Though the interaction energies of LPV, RTV, and NFV are comparable to each other, ulteriorly hinting to the binding pattern, their binding properties are different (Figure 4). Hence, we separately extracted the docked complex structure of the last frame of each 100 ns MD simulation to analyze the similarities and differences of the five binding modes (Figure 3).

### 2.2. Hydrogen Bond and Salt Bridge Interactions

The three-dimensional structure of SARS-CoV-2 Mpro in complex with ligand N3 indicated that the catalytic residues His41 and Cys145 should play important roles in the binding processes of substrates, and the interaction between ligands and residues His41 or Cys145 may induce the inhibiting effects. According to our simulation results (Table 1), in the SARS-CoV-2 Mpro-DRV complex, there are four hydrogen-bond interactions (Gln192 (0.230 nm), Met165 (0.240 nm), Thr26 (0.041 nm, 0.040 nm)). The two hydrogen bonds formed by DRV and Thr26 are relatively stable with the distances approximating 0.041 and 0.040 nm, respectively. In addition, residues His41, Cys145, Pro168, and Met49 have hydrophobic interactions with DRV. In the SARS-CoV-2 Mpro-LPV complex (Table 1), there exist 1 salt bridge (Glu166), 4 hydrogen bonds (Ser46 (0.290 nm), Glu166 (0.260 nm), Asn142 (0.310 nm), Gly143 (0.260 nm)), and 1 hydrophobic interaction (His41). Within the SARS-CoV-2 Mpro-NFV complex (Table 1), there are 1 salt bond (Glu166), 3 hydrogen bonds (Thr190 (0.250 nm), Met49 (0.260 nm), His164 (0.180 nm)) and 1 hydrophobic interaction (His41). There are 7 hydrogen bonds in the SARS-CoV-2 Mpro-RBV complex (Gln192 (0.280 nm), Thr190 (0.220 nm), His164 (0.230 nm), Met49 (0.290 nm), Arg188 (0.290, 0.250, and 0.240 nm)) (Table 1). Meanwhile, RTV has 1 salt bond (His41), 3 hydrogen bonds (Thr25 (0.280 nm), Gln189 (0.220 nm), His41 (0.230 nm)), and 2 hydrophobic interactions (His41 and Met49) with SARS-CoV-2 Mpro (Table 1). It is not hard to see some common points of these 5 binding poses: (1) LPV, NFV, RTV, and DRV all exhibit hydrophobic interactions with at least one of the catalytic residues His41 and Cys145, while RTV further forms salt bridge and hydrogen bonding interactions with residue His41; (2) in addition to the two key residues of His41 and Cys145, several other active-site residues of Mpro also take part in the hydrogen bond interactions, such as Thr25, Met49, Met165, Glu166, and Gln189; (3) in the SARS-CoV-2 Mpro-LPV and SARS-CoV-2 Mpro-NFV complexes, the ligands LPV and NFV all have the salt bridge interactions with residues Glu166 of Mpro, while RTV forms a salt bridge with residue His41. The formation of salt bonds facilitates the stable binding of ligands and receptors.

### 2.3. Binding Pocket Analysis

**Figure 5:**
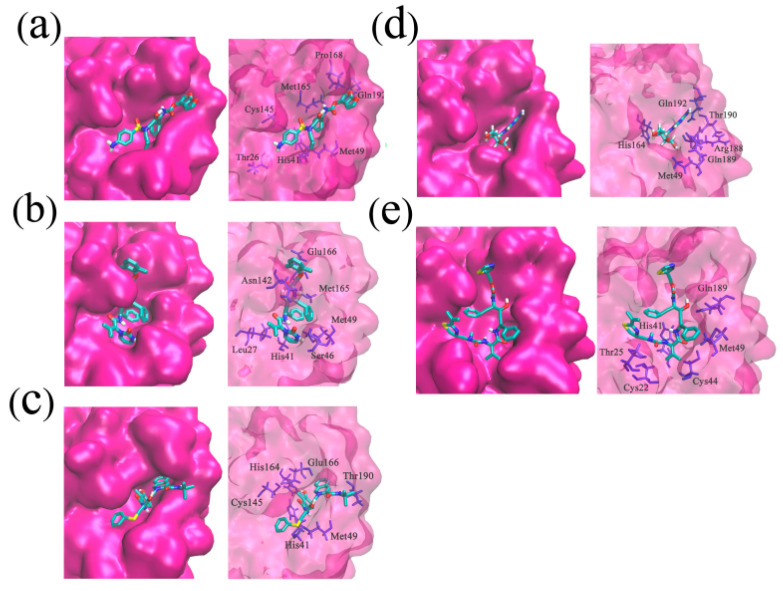
The key interactions at the active sites of the representative conformations of SARS-CoV-2 Mpro-DRV/LPV/NFV/RBV/RTV complexes with equilibrium stabilization. The interactions derived from the representative conformation of SARS-CoV-2 Mpro-DRV complex (**a**), SARS-CoV-2 Mpro-LPV complex (**b**), SARS-CoV-2 Mpro-NFV complex (**c**), SARS-CoV-2 Mpro-RBV complex (**d**), and SARS-CoV-2 Mpro-RTV complex (**e**) obtained from the MD trajectories. Many critical residues, e.g., Gln 192, Cys145, His41, His164, and Met49, are involved in the binding interactions. Residue interactions with ligand are colored in silver.

**Figure 6:**
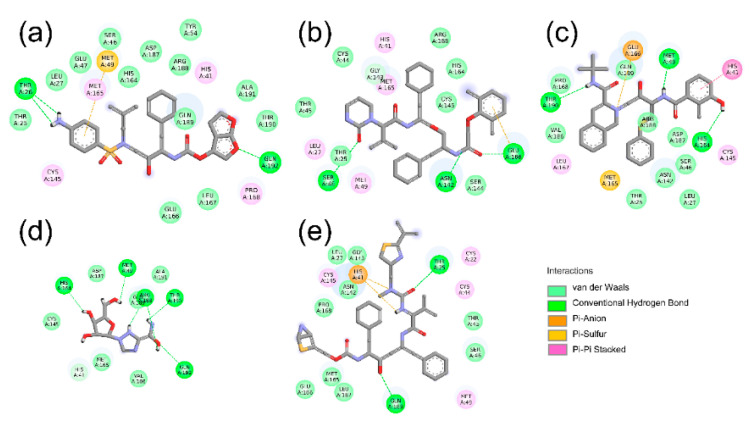
The interactions of SARS-CoV-2 Mpro-DRV/LPV/NFV/RBV/RTV complexes displayed as 2D image. In SARS-CoV-2 Mpro-DRV complex (**a**), Met49 with DRV forms a Pi–sulfur interaction and Thr26 with DRV forms two hydrogen bonds; in SARS-CoV-2 Mpro-LPV complex (**b**), the side chain of Glu366 with LPV forms a Pi–anion interaction, and Asn142, Ser46, and Glu166 are involved in three hydrogen bond interactions; in SARS-CoV-2 Mpro-NFV complex (**c**), the catalytic site His41 with NFV has a stable Pi–Pi stacked; in SARS-CoV-2 Mpro-RBV complex (**d**), two Van der Waals forces were produced in Gln189 and Thr190 with RBV, and four Hydrogen bonds were formed between His164, Gln192, Met49, Arg188, and RBV; in SARS-CoV-2 Mpro-RTV complex (**e**), we can see two Pi–anion interactions and hydrogen bonds. DRV/LPV/NFV/RBV/RTV are shown as the stick models. C, O, and N atoms are colored by gray, red, and blue, respectively. Hydrogen bonds and electrostatic interactions that help to lock the inhibitor are shown in green and orange dashed lines, respectively.

The substrate binding pocket of Mpro is located inside the cleft between domain I and domain II. In particular, the sub-binding sites S1, S2, and S4 of Mpro are highly conserved among all coronaviruses (Figure 4). Therefore, small molecules targeting these regions are supposed to have the broad-spectrum curative effect. As shown in Figure 5 and Figure 6, the binding location of DRV is distributed in the sub-binding site S4. The dimethylphenoxy group of LPV extends into the hydrophobic pocket of S1 and the two benzene rings of the side chains extend into the hydrophobic pocket of S4. At the same time, its diazacyclohexanone forms a hydrogen bond with residue Met49 in the S2 site. The *N*-tert-butyl decahydroisoquinoline of NFV binds well to the hydrophobic pocket of S4.

### 2.4. Stability Analysis of Docked Complexes

**Figure 7:**
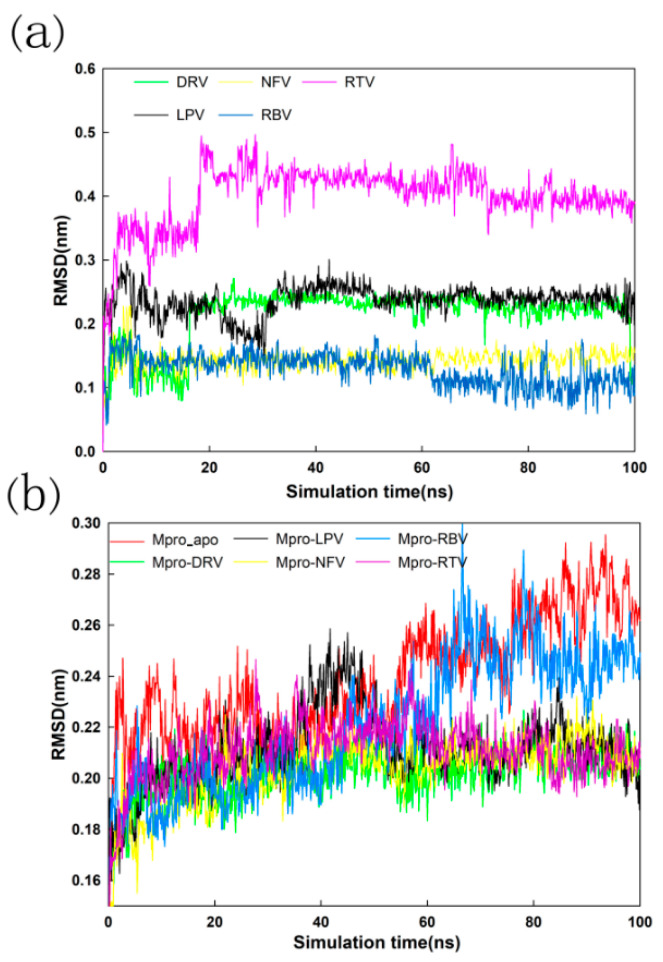
Variation in ligand positional RMSD (**a**) and backbone-atom RMSD (**b**) and docked complexes during the 100 ns MD simulations.

A 100 ns MD simulation was performed on DRV-Mpro, LPV-Mpro, NFV-Mpro, RBV-Mpro, and RTV-Mpro complexes and a Mpro-Apo protein not bound to small-molecule drugs. Preliminary analysis of the MD trajectory was carried out to check the structural stability and fluctuation of these small molecules and the protease Mpro complex according to the RMSD and RMSF results. The RMSD values of the DRV-Mpro, LPV-Mpro, NFV-Mpro, and RTV-Mpro complexes remained almost unchanged at 0.210 nm, which clearly shows that the complex structures of these four complexes are stable. The RMSD curve of the Mpro-apo and RBV-Mpro complexes deviated from the other four complexes at 50 ns, and gradually converged at 80 ns, but the RMSD value was much higher than the other complexes. In general, the structure of the complex with the small-molecule group is stable compared to that of the unbound molecule group. The combination of LPV, NFV, and RTV makes the Mpro more stable. At the same time, the RMSD values of small molecules in the complexes were also analyzed and compared. As shown in Figure 7, all five small molecules begin to converge and reach a stable value at 20 ns, indicating that the small molecules are stably fixed in the pocket, which bind to the Mpro from 20 ns. Due to the relatively small structure of small ligand molecules NFV and RBV, the RMSD value is at a small value. In addition, the RBV molecule appears to converge, as shown in Figure 7a, but the RBV-Mpro complex continues to elevate in Figure 7b. We suspect that domain III of Mpro is responsible for the conformational change, as indicated by the superimposition of the 90 ns conformation with the initial (0 ns) conformation from the MD trajectories of RBV-Mpro (RMSD = 0.180 nm). The differences of two conformations mainly occur in domain III of Mpro, which is far from the catalytic active site of Mpro.

**Figure 8:**
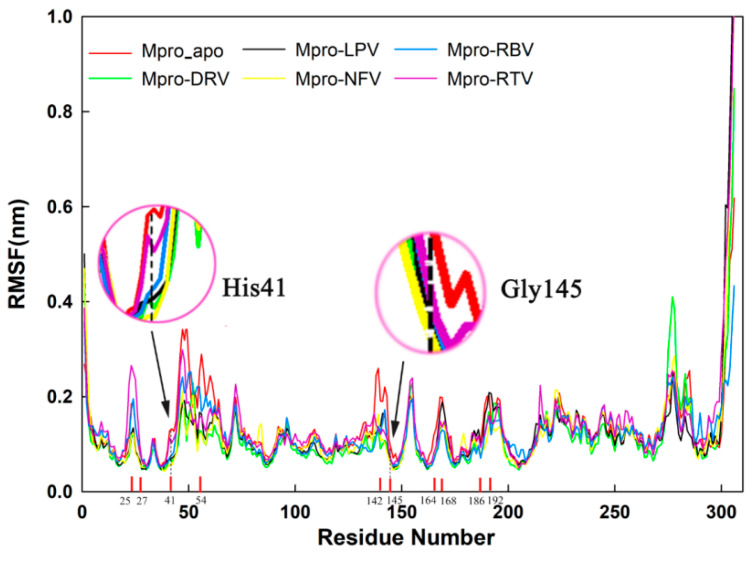
Determination of RMSF of unbound Mpro (Mpro_apo) and Mpro-DRV/LPV/NFV/RBV/RTV complexes. The RMSF values for Mpro (unbound) and Mpro-DRV/LPV/NFV/RBV/RTV complex were estimated from each 100 ns MD trajectory. The RMSF values of two catalytic residues were then plotted separately with red circles, and the main Mpro ligand pocket for five small molecules were labeled with smaller numbers.

As shown in Figure 8, all six systems (Mpro-apo, DRV-Mpro, LPV-Mpro, NFV-Mpro, RBV-Mpro, and RTV-Mpro) exhibit similar fluctuation patterns indicated by the time evolutions of RMSF. In the other five systems, the RMSF values of the two catalytically active sites and surrounding residues of Mpro are lower than the case of apo Mpro, and the same motion can be seen at other residues of the Mpro binding site. These results indicate that the combination of small molecules further stabilized the conformation of protease Mpro, especially the conformation of the catalytic center (Table 2 and Figure 8), although the RMSF fluctuation patterns show small differences between the two active sites.

### 2.5. MM–GBSA and Energy Decomposition

To gain insight into the inhibitory potential of five small-molecule drugs on Mpro, their binding free energy was calculated from the 100 ns MD trajectory by the MM/GBSA method. The MM–GBSA free energy values of DRV-Mpro, LPV-Mpro, NFV-Mpro, RBV-Mpro, and RTV-Mpro are 14.22 ± 49.00, −116.72 ± 32.90, −96.86 ± 63.66, 45.69 ± 49.64, and −103.55 ± 55.82 kJ/mol, respectively. It can be clearly seen that the LPV-Mpro, NFV-Mpro, and RTV-Mpro complexes show a higher binding free energy than the DRV-Mpro and RBV-Mpro complexes (Table 3), consistent with the interaction energy results observed in Section 2.1. Hydrogen-bonding or hydrophobic interactions between the ligands and the catalytic active site, as well as the salt bridge effect with other key sites, may be the key factors for the higher MM–GBSA value of the three complex systems, especially the salt bridge interaction. In accordance with our simulations, the binding free energies of the five compounds with Mpro decrease in the order of LPV > RTV > NFV > DRV > RBV. On the whole, the sorting is consistent with the reported in vitro enzymatic analysis and cell-based assay [25,26], except for the overmuch estimated value of LPV, which might come down to the inaccuracies of force fields and the polarity of the studied compounds [27]. Judging from the contribution of the four energies (Δ*E_vdw_,* Δ*E_ele_,* Δ*G_GB_,* and Δ*G_SA_*), Van der Waals components (Δ*E_vdw_* + Δ*G_SA_*) play a positive role in maintaining the stability of the complexes.

**Figure 9:**
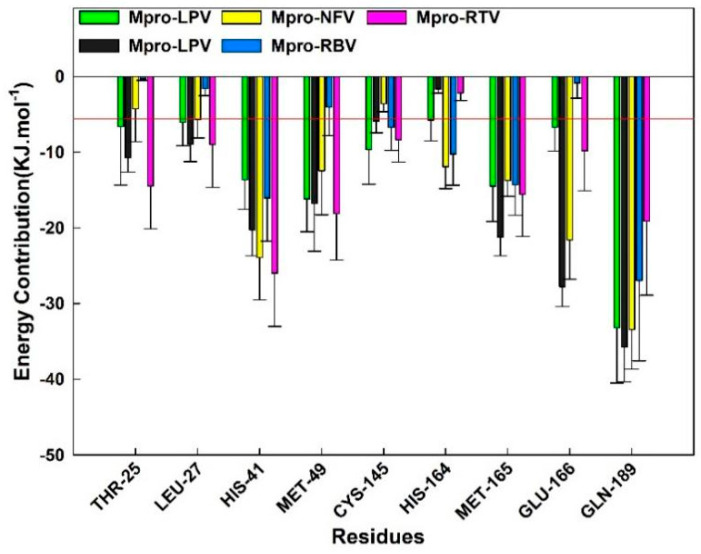
Binding free energy (*∆G_bind_*) contributions of Mpro-DRV/LPV/NFV/RBV/RTV complexes superposed residues in binding pockets. The residues contribution exceeding −5.0 kJ/mol to the binding free energy are marked with red baseline.

In order to estimate the specific contribution of a single residue to the binding free energy, we made overlaps of the binding pockets of protease Mpro in the five systems and calculated the free energy contribution of the overlapping residues. As shown in Figure 9, the contributions of residues Thr25, Leu27, Met49, and Cys145 in the DRV-Mpro, LPV-Mpro, NFV-Mpro, and RTV-Mpro complexes are better than −5.0 kJ/mol, which is conducive to the combination of small molecules and protease Mpro, and in the RBV-Mpro system, the energy decomposition value of these residues is greater than −5.0 kJ/mol. This difference may be the reason for the weak binding of RBV to Mpro. In addition, the contributions of the three residues His41, Met165, and Gln189 in the five systems are all less than the cutoff value, which may play a positive role in the stability of the complex structure.

### 2.6. Computational Alanine Scanning (CAS)

Key amino acid analysis can be analyzed by computational alanine scanning (CAS). CAS can evaluate the importance of the amino acid in the interaction process by calculating the energy change between the receptor and the ligand caused by the replacement of a specific amino acid with alanine. If this energy change (ΔΔ*G_mut_*) > 2.0 kJ/mol, it indicates that this amino acid plays an important role in promoting the binding of the receptor and the ligand.

**Figure 10:**
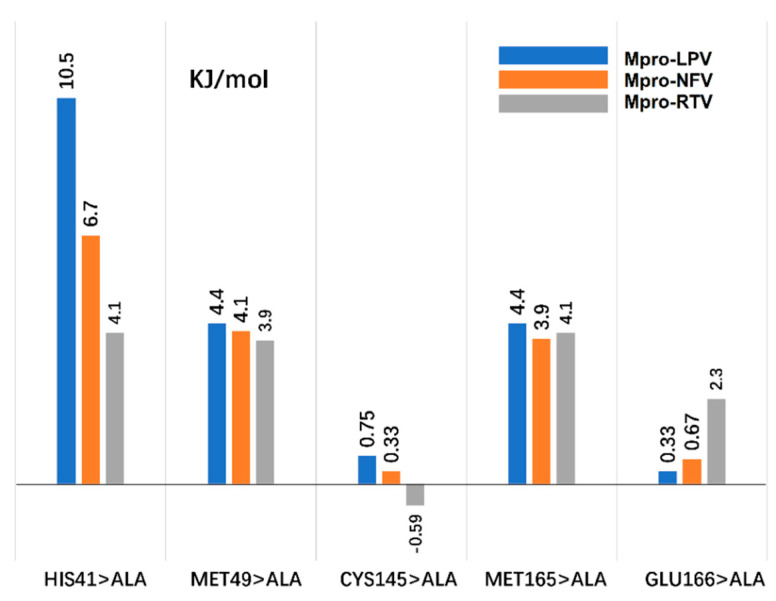
Computational alanine scanning (CAS) analysis with five key points His41, Met49, Cys145, Met165, and Glu166. The three systems of Mpro-LPV, Mpro-NFV, and Mpro-RTV use blue, orange, and gray bar charts, respectively.

According to the results of the residue contribution analysis in the MM–GBSA, for the three systems of LPV, NFV, and RTV, we selected five key points, including His41, Met49, Cys145, Met165, and Glu166 for CAS research, among which His41 and Cys145 are Mpro catalytic residues (Figure 10). Detailed parameters of CAS are displayed in our previous work [28].

In the Mpro-LPV system, we found three key points (His41 > Ala, Met49 > Ala, and Met165 > Ala). The mutation energy change can reach 10.5, 4.4, and 4.4 kJ/mol. Similarly, in the Mpro-NFV system, the energy changes of these three key point mutations were 6.7, 4.1, and 3.9 kJ/mol, respectively. The observed energy changes may be attributed to the damage of the hydrophobic pocket composed of His41, Met49, and Met165 of Mpro, which is crucial to provide a hydrophobic environment for the alkyl of LPV in the initial Mpro-LPV complex (Figure 5b). In the initial Mpro-NFV complex, His 41 is involved in forming a π–π interaction with NFV. The other hydrogen bond is formed by Met49 of Mpro with H71 of NFV (Figure 5c), which are critical to enhance ligands binding. These interactions are disrupted in this region upon the mutation of residues. This shows that these three key points play a key role in the interaction of these two small molecules with Mpro. However, in the Mpro-LPV system, we found that, in addition to the above three key points, the Glu166 > Ala mutation also had a greater impact on the combination of LPV. In this system, the GluA166 side chain forms a salt bridge with C42 of LPV; a salt bridge between amino acid residues of a single locus plays an important role in complex interaction. The salt bridge is absent in the Glu166>Ala mutation system, which may be attributed to the energy change (ΔΔ*G_mut_*) > 2.0 kJ/mol, indicating that Glu166 played a certain role in the process of combining LPV and Mpro, which is consistent with the above analysis of hydrogen bond formation and amino acid contribution.

Furthermore, we found that the binding mechanisms of HIV protease inhibitors DRV, LPV, NFV, and RTV with Mpro are different from those mentioned in recent works [29,30]. For example, the natural product compounds SN00293542 and SN00382835 have comparatively different binding profiles, except the similar interactions with His41 and Met49 of Mpro [29]. Overall, the modes of action mentioned in our simulations show some differences between these HIV protease inhibitors and other compounds, and this difference in mechanism may provide a new direction for anti-SARS-CoV-2 drug design [30].

### 2.7. Pharmacophore Model Analysis

**Figure 11:**
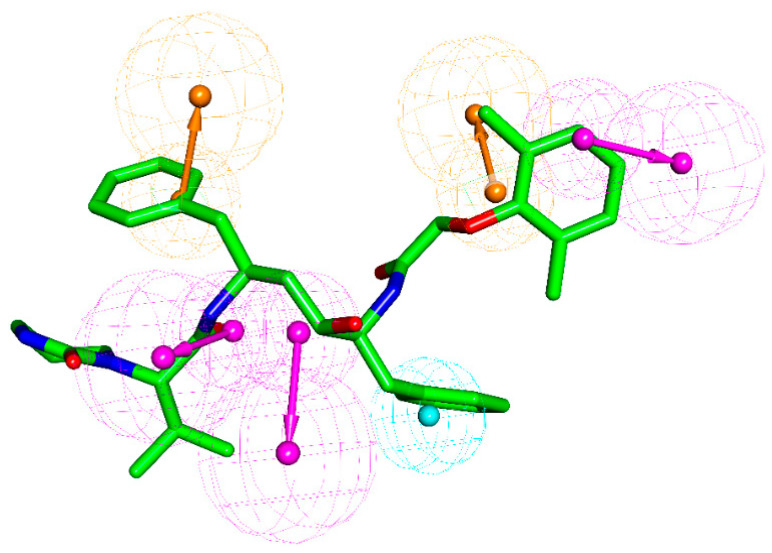
Pharmacophore features of the selected Mpro protease inhibitor. Hydrophobic group, hydrogen-bond donor, and aromatic ring are colored in light-blue, magenta, and brown, respectively. LPV is shown in stick model without H atoms. The five selected ligands were used to derive the common feature pharmacophore by using the “creating and validating a common feature pharmacophore” protocol of the Discovery studio (DS) software package, with the validating alignments of LPV and RBV.

By analyzing the pharmacophore properties of DRV, LPV, NFV, and RTV and the relationship between structure and Mpro interaction energy, we found that inhibitors with a better protein binding capacity should have two aromatic rings (ring aromatic group, brown), three hydrogen bond donors (hydrogen bond donor, magenta), and a hydrophobic group (hydrophobic group, light-blue), see Figure 11. According to this pharmacophore model, LPV should have a tolerable protease binding ability, which is consistent with the interaction and MM–GBSA analysis results. Due to of the lack of hydrogen bond donors, the binding affinity of NFV and RTV to Mpro protease is weak. Although DRV is relatively matched with this pharmacophore, its furan ring causes the poor matching of DRV. Meanwhile, RBV has the lowest matching with Mpro’s active cavity, which lacks an aromatic ring, hydrogen bond donor, and hydrophobic group. Based on the structure, electrostatic, hydrophilic, and hydrophobic properties of this pharmacophore and the active cavity of Mpro, for the rational drug design of anti-SARS-CoV-2 Mpro agents, groups might be suggested with both positive and hydrophobic properties, such as *N*-acetamido. In addition, considering the combination of aromatic rings, adding hydrogen bond donors and hydrophobic groups is the direction of COVID-19 drug design.

## 3. Materials and Methods

### 3.1. Structure Preparation

The crystal structure of the SARS-CoV2 main protease in complex with inhibitor N3 (PDB: 6LU7) was retrieved from the RCSB PDB database [31]. All the hetero-atoms of the nonprotein part were suppressed. At physiological pH, missing hydrogen atoms were added based on the expected charge distributions of amino acids [24]. Then, the charmm27 force field was used in the Generalized Born with a simple Switching (GBSW) solvent model. The energy minimizations were performed by a 1000-step steepest descent minimization, followed by conjugate gradient minimization, until converging to 0.40 kJ·mol^−1^·nm^−1^. All of the above processes were performed on the Discovery studio (DS) Charmm module [32].

The geometries of four commercial HIV protease inhibitors (RBV, LPV, NFV, and RTV) were obtained from 3BVB, 1MUI, 2PYM, and 1RL8, respectively. The broad-spectrum antiviral drug RBV was obtained from the crystal structure of RNA polymerase with mouse Norwalk virus (PDB: 5AXD). The atom structures and partial atomic charges of the five ligands were then handled by the “Minimize Ligands” tools in Discovery Studio software using the CHARMM force field, with a convergence criterion of 0.40 kJ·mol^−1^·nm^−1^ [32].

### 3.2. Docking and MD Simulations

In accordance to the previous reports [33,34,35], the docking process was performed using the cDocker algorithm [36], with the features for its grid-based method that the residues are held rigid and the ligands are free to move. The CHARMm-based molecular dynamics (MD) scheme was used to dock ligands into a receptor binding site. The binding site sphere was assigned with a radius 1.0 nm sphere. Combining random rotations and the simulated annealing method, all the poses of the docked complexes were scored to evaluate the docking results. The optimal orientation of each ligand within Mpro was probed on the basis of interaction energies and geometrical matching qualities [37,38]. The more negative the interaction energies of the docking complex, the higher the degree of the match between the receptor and the ligand. Energy minimizations were performed, using the conjugated gradient method, until converging to 0.40 kJ·mol^−1^·nm^−1^. The topology file of a small-molecule ligand in complex was generated by executing the python script for further molecular dynamics simulation.

The energy-minimized docked complexes were sufficiently equilibrated by 100.0 ns MD simulations, using the GROMACS5.1.2 program [39,40] and Amber-99sb force field. Each system was placed in a box (size 7.2 nm × 10.0 nm × 7.2 nm), and the box was filled with TIP3P water molecules. Na^+^ and Cl^−^ counter-anions were placed to maintain electrical neutrality for the whole system (0.15 M of NaCl) [41]. These five systems were minimized with a Steepest Decent (SD) method to remove wrong contacts. The NPT and NVP ensemble was applied. The pressure and temperature were coupled at 1 bar and 310 K by using Parrinello–Rahman barostats and the V-rescale thermostat, respectively [42]. The particle-mesh Ewald (PME) method was applied to handle the long-range electrostatics [43]. The cutoff distances for long-range electrostatic and van der Waals interactions were set to 0.8 and 1.0 nm, respectively. The Linear Constraint Solver (LINCS) method was applied to constrain the covalent bonds involving hydrogen atoms [44]. Each MD trajectory contained 1000 conformations. The coordinates were saved every 10.0 ps, with a time step of 2.0 fs.

### 3.3. Binding Energy Calculation by MM–GBSA

The MD trace was also used to calculate the binding free energy using the molecular mechanics–generalized Born surface area (MM–GBSA) approach [45,46]. The MM–GBSA module, implemented in Amber Tools16, is a common method to evaluate the binding strength of ligands and receptors [47,48]. The basic principle is shown in the formula [49]:

Δ*G*_bind_ = Δ*E*_MM_ + ΔΔ*G*_sol_ − *T*Δ*S*

= Δ*E*_MM_ + Δ*G*_GB_ + Δ*G*_SA_ − *T*Δ*S*

= Δ*E*_vdw_ + Δ*E_ele_* + Δ*G*_GB_ + Δ*G*_SA_ − *T*Δ*S*

where Δ*G_bind_* is the binding free energy, Δ*E_MM_* is the energy difference in the molecule under vacuum, including electrostatic (Δ*E_ele_*) and van der Waals (Δ*E_vdw_*) interactions, and ΔΔ*G_sol_* is the solvation free energy difference, including polar solvation energy (Δ*G_GB_*) and non-Polar solvation energy (Δ*G_SA_*). The entropic contribution *(T*Δ*S)* is evaluated with the normal-mode method [45,46]. The dielectric constants of the solvent and solute are 80 and 1, respectively [24,50]. Each residue contribution for the binding affinity was also decomposed by the MM–GBSA method. The energy decomposition value of these residues more than −5.0 kJ/mol could be regarded as key contributors for binding.

### 3.4. Computational Alanine Scanning (CAS)

Alanine scanning analysis refers to replacing specific amino acids of protein molecules with alanine to study the importance of amino acids. As alanine has only one methyl group and does not affect the chiral structure of the protein, alanine scanning is often used to calculate the importance of replaced amino acids in protein–protein/ligand interactions. Computational alanine scanning (CAS) was used to perform alanine substitution operations on the molecular model of the protein, and then calculate the energy change of the protein–protein/ligand system caused by the substitution to study the importance of the target amino acid.

We used the Calculate Mutation Energy (Binding) module under the DS platform to perform CAS calculations [51]. The basic principle is shown in the formula:

∆∆*G*_mut_ = ∆*G*_bind (mutant)_ − ∆*G*_bind (wild-type)_

Among them, ∆*G*_bind (mutant)_ and ∆*G*_bind (wild-type)_ refer to the binding energy in mutant and wild-type systems, respectively. ∆∆*G*_mut_ is the difference in binding energy between wild-type and mutant.

The GBSW solvent model was adopted to consider the solvation effect, and the electrostatic terms were approximated by the sum of coulombic interactions and polar contributions to solvation energy. The van der Waals interaction energy, side-chain entropy term, and nonpolar surface-dependent term were also included in the energy function of the GBSW model.

## 4. Conclusions

This study used molecular docking calculations, MD simulations, MM–GBSA, and pharmacophore analysis to carefully study the interaction of these small molecules with SARS-CoV-2 Mpro. Our results have revealed that four HIV protease inhibitors DRV, LPV, NFV, and RTV can stabilize the structure of Mpro through the interactions with catalytic residues His41 and the other two key amino acids Met49 and Met165. In contrast, no such interaction was found in the broad-spectrum antiviral agent RBV with the lowest binding capacity to Mpro. The results of residual energy decomposition and the interaction between small molecules and Mpro showed that the formation of hydrogen and salt bonds plays a key role in the ligand binding processes. In addition to the interaction of LPV, NFV, and RTV with catalytic sites His41 of protease Mpro, residues at other active sites around the catalytic sites, such as Thr25, Leu27, Met49, Met165, Glu166, and Gln189, also play important roles in the stabilization of complex structures. At the same time, the results of these theoretical analyses are consistent with the existing experimental analysis results.

In summary, among the four HIV protease inhibitors, LPV, RTV, and NFV have more interactions among multiple elements of SARS-CoV-2 Mpro, because of their aromatic rings and hydrogen bond donors. Our results provide information for the interactions of HIV-1 protease inhibitors with SARS-CoV-2 Mpro, and a promising reference for the development of novel anti-SARS-CoV-2 agents. This research also provides inspiration to researchers and proposes a new direction for the molecular design of COVID-19 drugs. Based on this, and then using experimental verification to develop small-molecule drugs with real application value, it will contribute to the prevention and control of the COVID-19 epidemic.

## Figures and Tables

**Table 1:** Hydrogen bond and salt bridge interactions in the five selected docked complexes.

Complex	Donor and Acceptor	Category
SARS-CoV-2 Mpro-DRV	GLN192:H–DRV:O26	Hydrogen Bond
	MET165:HA–DRV:O10	Hydrogen Bond
	DRV:H39–THR26:O	Hydrogen Bond
	DRV:H40–THR26:O	Hydrogen Bond
	HIS41:Pi-Orbitals–DRV:Alkyl	Hydrophobic
	DRV:Pi-Orbitals–CYS145:Alkyl	Hydrophobic
	DRV:Pi-Orbitals–PRO168:Alkyl	Hydrophobic
	DRV:C15–MET49:Alkyl	Hydrophobic
SARS-CoV-2 Mpro-LPV	GLU166:OE1–LPV:C42	Electrostatic
	SER46:HG–LPV:O12	Hydrogen Bond
	GLU166:H–LPV:O38	Hydrogen Bond
	LPV:H78–ASN142:OD1	Hydrogen Bond
	GLY143:HA2–LPV:O20	Hydrogen Bond
	HIS41:Pi-Orbitals–LPV:Alkyl	Hydrophobic
SARS-CoV-2 Mpro-NFV	NFV:N7–GLU166:OE1	Electrostatic
	NFV:H56–THR190:O	Hydrogen Bond
	NFV:H71–MET49:SD	Hydrogen Bond
	NFV:H77–HIS164:O	Hydrogen Bond
	HIS41:Pi-Orbitals–NFV:Pi-Orbitals	Hydrophobic
	NFV:C33–CYS145:Alkyl	Hydrophobic
	HIS41:Pi-Orbitals–NFV:C33	Hydrophobic
	NFV:Pi-Orbitals–LEU167:Alkyl	Hydrophobic
	NFV:Pi-Orbitals–MET49:Alkyl	Hydrophobic
SARS-CoV-2 Mpro-RBV	GLN192:HE21–RBV:O5	Hydrogen Bond
	RBV:H18–THR190:O	Hydrogen Bond
	RBV:H25–HIS164:O	Hydrogen Bond
	RBV:H29–MET49:SD	Hydrogen Bond
	RBV:H6–ARG188:O	Hydrogen Bond
	RBV:H7–ARG188:O	Hydrogen Bond
	RBV:H14–ARG188:O	Hydrogen Bond
SARS-CoV-2 Mpro-RTV	RTV:N39–HIS41:Pi-Orbitals	Electrostatic
	THR25:HG1–RTV:O41	Hydrogen Bond
	GLN189:HE21–RTV:O22	Hydrogen Bond
	RTV:H77–HIS41:Pi-Orbitals	Hydrogen Bond
	RTV:C50–HIS41:Pi-Orbitals	Hydrophobic
	RTV:Pi-Orbitals–MET49:Alkyl	Hydrophobic

**Table 2:** The RMSF value of the catalytic center of the Mpro-DRV/LPV/NFV/RBV/RTV complex and Mpro_apo.

Complex	His41 (nm)	Gly145 (nm)
Mpro_apo	0.128 ± 0.009	0.090 ± 0.007
Darunavir	0.070 ± 0.010	0.056 ± 0.007
Lopinavir	0.067 ± 0.012	0.063 ± 0.010
Nelfinavir	0.057 ± 0.008	0.057 ± 0.012
Ribavirin	0.072 ± 0.009	0.067 ± 0.009
Ritonavire	0.113 ± 0.013	0.066 ± 0.011

**Table 3:** The binding energies (ΔE), entropies (ΔS), and free energies (ΔG) for the Mpro-DRV/LPV/NFV/RBV/RTV complexes (unit in kJ/mol).

Complex	ΔE_vdw_	ΔE_ele_	ΔG_GB_	ΔG_SA_	TΔS _a_	ΔGS _b_
Darunavir (DRV)	−276.98 ± 17.49	−337.44 ± 36.65	558.19 ± 35.31	−27.03 ± 1.34	−97.48 ± 14.94	14.22 ± 49.00
Lopinavir (LPV)	−260.66 ± 12.51	−75.65 ± 15.94	134.10 ± 14.64	−30.63 ± 1.42	−116.12 ± 16.42	−116.72 ± 32.90
Nelfinavir (NFV)	−318.70 ± 35.94	−382.42 ± 56.90	542.03 ± 62.51	−26.61 ± 2.85	−88.83 ± 25.89	−96.86 ± 63.66
Ribavirin (RBV)	−122.93 ± 15.10	−75.65 ± 32.34	172.46 ± 27.15	−16.28 ± 1.30	−88.07 ± 21.02	45.69 ± 49.64
Ritonavire (RTV)	−311.54 ± 42.89	−13.97 ± 35.69	151.96 ± 33.18	−31.34 ± 2.22	−101.34 ± 29.29	−103.55 ± 55.82

_a_ The entropic energies are calculated with a generalized−Born solvent model (nmode_igb = 1); _b_ The entropic contributions are included in the binding free energies (ΔGS).

## Data Availability

All data generated or analyzed during this study are included in this published article.

## Sample Availability

Samples of the compounds are available from the authors.
